# PCBs May Impede IVF Success: Failed Embryo Implantation Linked to Exposure

**DOI:** 10.1289/ehp.119-a307a

**Published:** 2011-07-01

**Authors:** Julia R. Barrett

**Affiliations:** Julia R. Barrett, MS, ELS, a Madison, WI–based science writer and editor, has written for *EHP* since 1996. She is a member of the National Association of Science Writers and the Board of Editors in the Life Sciences.

Although banned from production in the United States and other developed countries in the late 1970s, polychlorinated biphenyls (PCBs) remain widespread environmental contaminants found in measurable amounts in the general population. These compounds have been linked to longer time to pregnancy and increased pregnancy loss in both epidemiologic and experimental research. A new study pinpoints failed embryo implantation in the uterus as a possible mechanism for these outcomes [*EHP* 119(7):1010–1016; Meeker et al.].

Between 1994 and 2003, 2,350 Boston-area couples undergoing *in vitro* fertilization (IVF) were recruited for a study of predictors of IVF outcomes. Study participants included women who underwent embryo transfer and did not become pregnant, those who had a positive pregnancy test but no fetal development (known as a “chemical pregnancy”), those who became pregnant and had a miscarriage (pregnancy loss before 20 weeks), and those who had a live birth. The study included 765 women and 827 IVF cycles resulting in 229 implantation failures, 177 chemical pregnancies, 124 miscarriages, and 297 live births.

Prior to their first IVF cycle, the women provided blood samples for measurement of PCBs, including 57 individual congeners. IVF outcome was assessed against concentrations of three individual PCB congeners (PCBs 118, 138, and 153), all PCBs combined (ΣPCBs), and three separate PCB groupings (groups 1–3) based on structure and biological activity.

None of the individual PCBs or PCB groupings were associated with chemical pregnancy or spontaneous abortion. However, significant trends emerged for failed implantation and reduced odds of a live birth. PCB 153 and ΣPCBs were both dose-dependently associated with failed implantation, with the odds doubled in the highest quartile of exposure (> 0.34 ng/g and > 1.81 ng/g, respectively) versus the lowest (≤ 0.16 ng/g and ≤ 0.32 ng/g, respectively).

Women with PCB 118 and group 3 congener concentrations above the lowest quartile also were more likely to have a failed implantation than women with the lowest exposures, although the associations did not increase with each increase in dose. Odds of a live birth were lower for women in the highest versus lowest quartiles of PCB 153 and ΣPCB exposure, while PCB 118 and group 2 and 3 congeners were associated with smaller nonsignificant reductions in live births.

The study has several strengths, including its prospective design and PCB measurement at the start of attempted pregnancy. However, unknown confounding factors, additional reproduction-related end points for PCB toxicity, relevance of these results to women not undergoing IVF, and a lack of data on male factors are gaps that need to be filled in future studies.

